# Short chain fatty acids prime colorectal cancer cells to activate antitumor immunity

**DOI:** 10.3389/fimmu.2023.1190810

**Published:** 2023-05-25

**Authors:** Courtney Mowat, Jasmine Dhatt, Ilsa Bhatti, Angela Hamie, Kristi Baker

**Affiliations:** ^1^ Department of Oncology, University of Alberta, Edmonton, AB, Canada; ^2^ Department of Medical Microbiology and Immunology, University of Alberta, Edmonton, AB, Canada

**Keywords:** colorectal cancer, antitumor immunity, microbiota, short chain fatty acid (SCFA), microsatellite instability, HDAC

## Abstract

**Introduction:**

Colorectal cancer (CRC) is a leading cause of death worldwide and its growth can either be promoted or inhibited by the metabolic activities of intestinal microbiota. Short chain fatty acids (SCFAs) are microbial metabolites with potent immunoregulatory properties yet there is a poor understanding of how they directly regulate immune modulating pathways within the CRC cells.

**Methods:**

We used engineered CRC cell lines, primary organoid cultures, orthotopic in vivo models, and patient CRC samples to investigate how SCFA treatment of CRC cells regulates their ability to activate CD8+ T cells.

**Results:**

CRC cells treated with SCFAs induced much greater activation of CD8+ T cells than untreated CRC cells. CRCs exhibiting microsatellite instability (MSI) due to inactivation of DNA mismatch repair were much more sensitive to SCFAs and induced much greater CD8+ T cell activation than chromosomally instable (CIN) CRCs with intact DNA repair, indicating a subtype-dependent response to SCFAs. This was due to SCFA-induced DNA damage that triggered upregulation of chemokine, MHCI, and antigen processing or presenting genes. This response was further potentiated by a positive feedback loop between the stimulated CRC cells and activated CD8+ T cells in the tumor microenvironment. The initiating mechanism in the CRCs was inhibition of histone deacetylation by the SCFAs that triggered genetic instability and led to an overall upregulation of genes associated with SCFA signaling and chromatin regulation. Similar gene expression patterns were found in human MSI CRC samples and in orthotopically grown MSI CRCs independent of the amount of SCFA producing bacteria in the intestine.

**Discussion:**

MSI CRCs are widely known to be more immunogenic than CIN CRCs and have a much better prognosis. Our findings indicate that a greater sensitivity to microbially produced SCFAs contributes to the successful activation of CD8+ T cells by MSI CRCs, thereby identifying a mechanism that could be therapeutically targeted to improve antitumor immunity in CIN CRCs.

## Introduction

There is an increasing appreciation for the role played by the human microbiome in driving health and disease (1, 2). A primary driver of both the helpful and harmful effects of the microbiota are metabolic byproducts such as short chain fatty acids (SCFAs) generated from the metabolism of dietary fiber (3, 4). SCFAs such as butyrate, propionate and acetate act as fuel for intestinal epithelial cells and promote critical homeostatic functions in the intestine. Consistent with this, SCFAs generally exert a protective effect against colorectal cancer (CRC) by decreasing tumor cell proliferation and increasing differentiation (3, 5, 6). This is primarily due to the function of SCFAs as histone deacetylase (HDAC) inhibitors that can block cell cycle progression and promote the induction of apoptosis (7, 8). While this would generally be expected to decrease the amount of DNA damage in cancer cells, numerous reports indicate that SCFAs can promote the accumulation of DNA damage in CRC cells by interfering with DNA repair mechanisms (7, 9–12). It is thus likely that the antitumorigenic effects of SCFAs involve more complex mechanisms extending beyond the tumor cells themselves. This may be especially significant in the case of CRC cells that have an underlying DNA repair defect, such as the microsatellite instability high (MSI) CRC subset that is known for its high immunogenicity.

In addition to acting directly on the intestinal epithelium, SCFAs play a key anti-inflammatory role in regulating local and systemic immune cells (13, 14). This includes promoting the production of antimicrobial compounds, inhibition of neutrophils and macrophages, activation of regulatory T cells, and induction of tolerogenic properties in dendritic cells (14). Since inflammation is a potent driver of tumor progression, these effects are likely to contribute to the antitumor effects of SCFAs. However, tumor-targeted T cell responses are a critical component of antitumor immunity and are increasingly recognized as an important contributor to the efficacy of many cancer treatments (15–18). Suppression of such responses specifically by the SCFA butyrate could thus contribute to tumor progression and have a very detrimental effect on treatment outcome. It is thus critical to better understand the dynamic relationship between the CRC cells, immune cells, and SCFAs.

Despite the direct influence of SCFAs like butyrate and propionate on either CRC cells or immune cells having been relatively well characterized, little is known about how SCFAs change immune-related processes within CRC cells. This is particularly important to understand given the architecture of the intestine where colonic epithelial cells will have far greater exposure to SCFAs produced by lumenal microbiota than will the underlying immune cells in the lamina propria. Thus, modulating intestinal epithelial cell immune properties, including those of CRC cells, may be the primary way that SCFAs regulate immune responses in the intestine and beyond. This study represents the first in depth exploration of how SCFAs modulate the antitumor immune response via their effects directly on CRC cells.

## Methods

### Cell culture and organoid generation

MC38 mouse CRC cells were originally purchased from Kerafast. The cells were stably transfected with an OVA-expressing plasmid and then MSI^OVA^ and CIN^OVA^ variants were generated by deleting *Mlh1* or mutating *Kras*, respectively, as described previously (16). The *Sting* knockdown variants of these cell lines were created with the pLKO.1 system using the shRNAs in **Table S1** or a scrambled control sequence (16, 19).

Murine organoids from colorectal tumors induced by repeated doses of azoxymethane (10 weekly doses of 10 mg/kg azoxymethane) were generated as described previously (16, 20, 21). In brief, tumors were dissociated for 1 h in DMEM with 2.5% FBS, 75 U/ml collagenase XI (SigmaAldrich), 125 µg/ml dispase II (SigmaAldrich). Following filtration, cells were plated at 500-1000 per well in growth factor reduced Matrigel (Corning) and cultured in basal crypt media (Advanced DMEM/F12containing 10% FBS, 2 mM glutamine, 10 mM HEPES, 1 mM N-acetylcystein, 1X N2 supplement, 1X B27 supplement, 10 mM nicotinamide, 500 nM A83-01, 10 µM SB202190, 50 ng/ml EGF) (ThermoFisher) mixed 1:1 with conditioned supernatant from L-cells expressing Wnt3a, R-spondin and noggin (ATCC #CRL-3276) (22).

Human organoids were generated from resected human CRC tumors that were collected in HBSS within 10 min of devitalization. The tumors were processed as described previously (21). In brief, tumors were dissociated in DMEM containing 2.5% FBS, 75 U/ml collagenase XI (SigmaAldrich), 125 µg/ml dispase II (SigmaAldrich) for 1 h at 37°C. Following filtration and extensive washing, 500-1000 cells per well were plated in growth factor reduced Matrigel (Corning) and cultured in basal crypt media (Advanced DMEM/F12containing 10% FBS, 2 mM glutamine, 10 mM HEPES, 1 mM N-acetylcystein, 1X N2 supplement, 1X B27 supplement, 10 mM nicotinamide, 500 nM A83-01, 10 µM SB202190, 50 ng/ml EGF) (ThermoFisher) mixed 1:1 with conditioned supernatant from L-cells expressing Wnt3a, R-spondin and noggin (ATCC #CRL-3276) (22, 23). All work with human samples was approved by the Health Research Ethics Board of Alberta Cancer Committee and carried out after obtaining informed patient consent.

Knockdown of *MLH1* in the primary MSI mouse and human organoids was achieved using lentiviral transduction as described previously using the pLKO.1 system (Addgene #10878) containing the shRNA sequences in **Table S1** (16, 19, 24, 25).

### SCFA stimulation

Cells were seeded 24 h (MC38 CRC) or 3 days (organoids) ahead of time and then treated with 50 mM butyrate, 50 mM propionate, or a combination of 50 mM butyrate and 50 mM propionate for the indicated times. In some experiments, cells were pretreated with the following reagents for 1 h before addition of the SCFAs: 100 mM BHB (SigmaAlrich), 10 μM H151 (SigmaAldrich), 1 µM trichostatin A (SigmaAldrich), 5 µg/ml anti-IFNGR (BioXcell), 100 U/ml IFNγ (RnD Systems).

### RNA isolation and qPCR

RNA was extracted using Trizol and reverse transcribed using the High-Capacity cDNA Reverse Transcription Kit (ThermoFisher). qPCR reactions were set up using the primers indicated in **Table S1** and POWRUP SYBR Master Mix (ThermoFisher). qPCR was performed on the QuantStudio6 real-time PCR system (Applied Biosystems).

### Protein isolation and western blotting

Protein was isolated in lysis buffer (50 mM Tris-HCl, 150 mM NaCl, 50 mM sodium pyrophosphate, 1 mM EDTA, 0.5% NP40, 1% Triton X-100) containing 1 mM sodium orthovanadate, and 1x protease inhibitor (SigmaAldrich) (16). Protein was quantified using a BCA protein assay kit (ThermoFisher). Equal amounts of protein was loaded per lane of SDS-PAGE gels and transferred to nitrocellulose membranes. The antibodies used are listed in **Table S2**. Bands were visualized using the ECL Prime Western Blotting Detection Reagent (GE Healthcare Amersham).

### Flow cytometry

Staining was performed using antibodies listed in **Table S2** at a 1:200 dilution as well as the Zombie Aqua viability stain (BioLegend). All intracellular staining was performed using the Foxp3 Transcription Factor Staining Buffer Set (eBioscience). Samples were acquired on CytoFlex S cytometer (Beckman Coulter) and data was analyzed using FlowJo software (BD Biosciences).

### Orthotopic mouse model

C57BL/6 wildtype mice originally were purchased from Charles River and maintained in the Cross Cancer Institute vivarium. OTI mice were purchased from The Jackson Laboratory. Male and female littermates between the age of 6-20 weeks old were used for all experiments. All animal work was approved by the Cross Cancer Institute’s Animal Care Committee.

Orthotopic CRC experiments were performed by injecting 1.5x10^5^ MC38 CRC cells in 50 µl PBS into the wall of the descending colon using a flexible needle (Hamilton) inserted through the working channel of a Wolfe endoscope and visualized via the ColoView imaging system (Storz) (16, 25). Tumors were harvested after 14-21 days and tissue samples were snap frozen.

### scRNAseq

scRNAseq was previously published by us on orthotopically grown MSI and CIN CRCs and deposited as dataset GSE178706 at the NCBI Gene Expression Omnibus (16). Gene signature expression analysis (GSEA) was performed as in the original publication to identify Gene Ontology (GO) signatures associated with each CRC subtype (16, 26).

### 16S rRNA sequencing

Fecal samples from each mouse had been collected at the time of tumor harvest. Each group contained 6 mice that were processed and sequenced individually and then pooled for the final analysis. Samples were lysed using 750 µl of lysis buffer (200 mM NaCl, 100 mM Tris pH 8.0, 20 mM EDTA, 20 mg/ml lysozyme (SigmaAldrich)) at 37˚C for 30 min. A blank tube was isolated to serve as a kit contamination control. Samples were resuspended in 85 µl 10% SDS in 30 μl of Proteinase K (20 mg/ml) (NEB) and incubated at 60˚C for 30 min. Samples were added to screwcap tubes with 300 mg of 1 mm beads and 500 µl phenol:chloroform:isoamyl alcohol (25:24:1) (Thermo Fisher Scientific) and beaten in a bead beater on high for 2 min, then spun at 10,000xg for 5 min. The aqueous layer was added to 500 µl of phenol:chloroform:isoamyl alcohol (25:24:1), vortexed, then spun at 14,000xg for 5 min and this step was repeated two more times before the final aqueous phase was precipitated with ethanol and 60 µl of 3 M sodium acetate (pH 5.2) at -20˚C for ≥ 1 hour. Samples were spun for 10 min at 14,000xg, and the pellets were dried and resuspended in Tris buffer (10mM, pH8.0). The DNA was then isolated using the QiaAmp Fast DNA Stool kit (Qiagen) and quantified using the QuantIT PicoGreen dsDNA Assay Kit (Thermo Fisher Scientific) prior to submission to Novogene for sequencing. The V3-V4 variable regions of the 16S ribosomal RNA (rRNA) gene was PCR amplified using specific barcoded primers (**Table S1**) possessing barcodes along with the Phusion^®^ High-Fidelity PCR Master Mix (New England Biolabs). The preparation of the library was done with the IonS5™XL Fragment Library Kit (Thermo Fisher Scientific) prior to sequencing. The data was analyzed in QIIME V1.7.0 (Quantitative Insights Into Microbial Ecology) and the reads compared with the Gold database using the UCHIME algorithm to obtain effective reads. Sequences with ≥ 97% similarity were assigned the same OTU using Uparse v7.0.1001. To annotate species at each taxonomic rank, Mothur software was performed against the SSUrRNA database of the SILVA database. MUSCLE v3.8.31 was used to get the phylogenetic relationship of all OTUs. Z-scores were calculated from the raw data generated by Novogene and used to compare taxa between samples. The data has been deposited at GenBank under the accession number PRJNA963222.

### Metabolomics analysis

Fecal samples from each mouse had been collected before tumor induction and at the time of tumor harvest. Tumor tissue samples had been snap frozen at the time of tumor harvest. Samples of each type were pooled into groups for processing and submitted to The Metabolomics Innovation Center (University of Alberta). Each post tumor fecal sample was normalized to its own baseline control. Metabolite abundance was then compared between groups and analyzed using a two-way ANOVA (CI > 95%) with Sidak’s multiple comparisons (Prism, GraphPad). To identify common metabolic pathways associated with each tumor type, the metabolites found to be significantly upregulated or downregulated in each condition were analyzed in the MetaboAnalyst platform (27, 28).

### Human CRC data

Human RNA sequencing data (Illumina HiSeq RNASeqV2) and Microbial Signatures (log-cpm) from the Colorectal Adenocarcinoma dataset from the TCGA Nature 2012 and TCGA PanCancer Atlas from The Cancer Genome Atlas were downloaded from cBioPortal for Cancer Genomics (https://www.cbioportal.org/) (29–31). Expression analysis was performed using the DESeq2 package in R (v3.0) (32).

### Statistical analysis

Prism (GraphPad) was used for statistical analysis. Gene expression analysis was processed by log2 transformation and resulting data evaluated for Gaussian distribution. Comparisons of two unpaired groups was made by two-tailed Student’s t-test for normal data, or Mann-Whitney for non-parametric tests. For three or more groups with two biological replicates each, two-way ANOVA or multiple t-test procedures were used as appropriate. *Post-hoc* analysis to correct for multiple comparisons during two-way ANOVA was performed using Tukey’s multiple comparison test. A two-sided probability (p) of alpha error less than 0.05 defined significance.

## Results

### SCFAs prime colorectal cancer cells to activate CD8+ T cells

**Figure 1:**
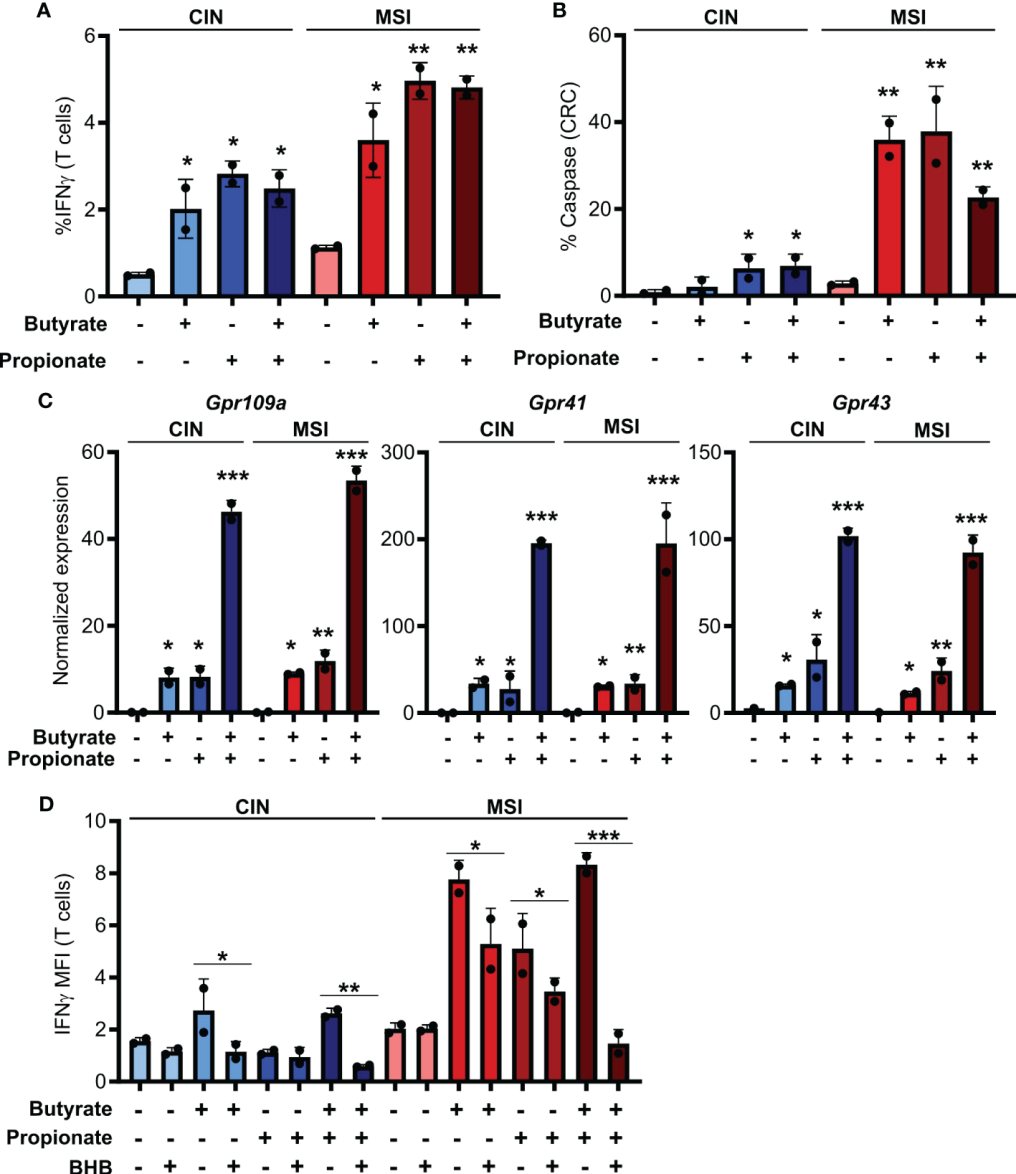
SCFAs increase the ability of CRCs to activate CD8+ T cells. **(A, B)** MSI^OVA^ and CIN^OVA^ CRC cells were pretreated for 24 h with 50 mM butyrate, 50 mM propionate or a combination of the two. SCFAs were washed off and OVA-specific OTI CD8+ T cells were then added and cocultured with the CRC cells for 24 h before measuring T cell IFNγ production **(A)** and T cell-mediated CRC killing **(B)**. **(C)** Expression of the main SCFA receptors was analyzed by qPCR in CRC cells stimulated for 24 h by 50 mM butyrate and/or propionate. **(D)** Cocultures were performed as in **(A)** with the inclusion of 100 mM BHB during the butyrate/propionate treatment. For all panels, n = 3 experimental repeats with 2 biological replicates per experiment. Representative graphs from a single experiment are shown. For **(A–C)**, relative to the untreated control: *p ≤ 0.05, **p ≤ 0.01, ***p ≤ 0.001. For **(D)** *p ≤ 0.05, **p ≤ 0.01, ***p ≤ 0.001.

To first test how SCFAs influence the immunogenicity of MSI and CIN CRCs, we generated OVA-expressing MSI^OVA^ and CIN^OVA^ clones of the MC38 mouse CRC cell line by knocking out *Mlh1* or by mutating *Kras*, respectively (16). We stimulated the different CRC variants with butyrate, propionate or a combination of the two for 24 h before removing these metabolites, adding OVA-specific CD8+ T cells from OTI transgenic mice and coculturing for 48h. In contrast to the well-known direct immunoregulatory effects of SCFAs, stimulating both MSI^OVA^ and CIN^OVA^ CRCs greatly increased their ability to activate OVA-specific OTI T cells and induce CD8+ T cell-mediated killing of the CRCs (**Figures 1A, B**). Notably, this effect was consistently stronger for the treated MSI^OVA^ CRC cells. This is consistent with our previous finding that not only are MSI CRCs are more immunogenic than CIN CRCs at baseline but they are also more responsive to the immune stimulating effects of the microbiota in their environment (16).

Butyrate and propionate act via two primary mechanisms. The first is binding to pleotropic surface receptors (GPR41, GPR43 and GPR109a) and activating downstream signaling (33, 34). The second is direct entry into the cytosol followed by binding to HDACs, leading to their inhibition (7, 8). To determine which of these mechanism accounts for the differential immunoregulatory effect of SCFAs on MSI and CIN CRCs, we looked at expression of the various receptors known to bind butyrate and propionate. We did not detect differential gene expression of these between our MSI^OVA^ and CIN^OVA^ CRC variants either at baseline or upon treatment of the cells (**Figure 1C**). To functionally examine the role of the receptors in CD8+ T cell activation, we repeated the stimulation above following pretreatment of the CRC cells with the GPR41 blocking agent beta-hydroxybutyrate (BHB) (35–37). This significantly suppressed OTI T cell activation by the treated CRCs, indicating that butyrate and propionate increase the immunogenicity of CRC cells via a GPR-dependent mechanism (**Figure 1D**).

### SCFA induce an IFNγ-dependent feedback loop between CRC and CD8+ T cells that upregulates CRC MHCI and amplifies T cell activation

**Figure 2:**
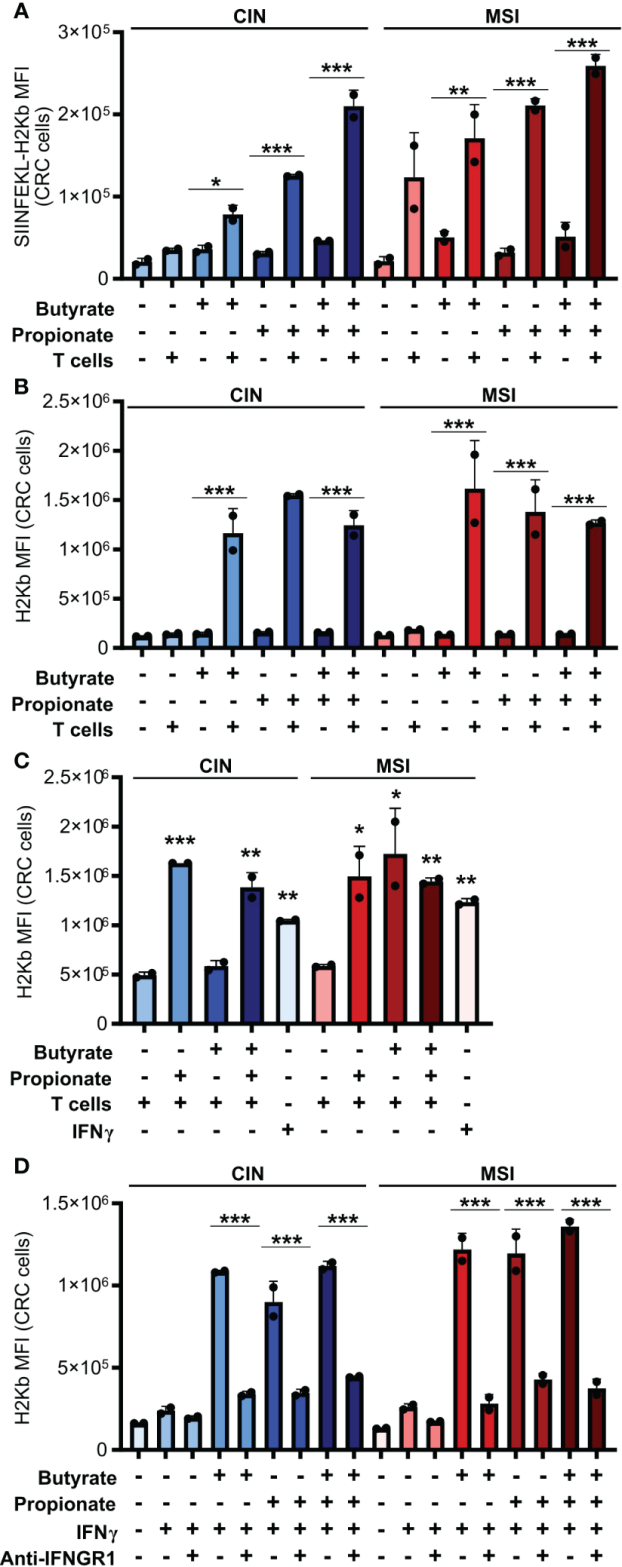
CD8+ T cell activation by SCFA-treated CRCs depends on an IFNγ-driven positive feedback loop that upregulates CRC MHCI. **(A, B)** MSI^OVA^ and CIN^OVA^ CRC cells were pretreated for 24 h with 50 mM butyrate, 50 mM propionate or a combination of the two. SCFAs were washed off the CRC cells and they were then cocultured or not with OVA-specific OTI CD8+ T cells for 24 h. Expression of SIINFEKL-H2Kb **(A)** or total H2Kb **(B)** was measured on the CRC cell surface by flow cytometry. **(C)** CRC cells were treated as in **(A)** but 100 U/ml IFNγ was added as indicated to wells without T cells. **(D)** CRC cells were treated with 50 mM butyrate/propionate for 24 h in the presence of 100 U/ml IFNγ and 5 μg/ml anti-IFNGR or an isotype control. For all panels, n = 3 experimental repeats with 2 biological replicates per experiment. Representative graphs from a single experiment are shown. For panels **(A, B, D)**: *p ≤ 0.05, **p ≤ 0.01, ***p ≤ 0.001. For panel **(C)**, relative to the untreated control: *p ≤ 0.05, **p ≤ 0.01, ***p ≤ 0.001.

In seeking to understand how butyrate and propionate were promoting the ability of CRC cells to stimulate antitumor immunity, we examined whether treatment with these metabolites increased presentation of the OVA antigen on MHCI on the CRC cell surface. Both butyrate and propionate increased the amount of surface MHCI bound to the SIINFEKL OVA epitope presented on the surface of CRC cells (**Figure 2A**). However, this only occurred with the SCFA-treated CRC cells that were subsequently cocultured with CD8+ T cells and not on CRC cells unexposed to T cells. We observed similar results for expression of total MHCI on the surface of CRC cells incubated with CD8+ T cells (**Figure 2B**). Since the SCFAs were washed away from the CRC cells before addition of the T cells, this cannot be explained by the effects of butyrate or propionate directly on the T cells. Instead, our data suggests that SCFA stimulation of CRC cells changes the outcome of their cross-talk with CD8+ T cells.

IFNγ is known to upregulate MHCI expression and, given that we had observed increased IFNγ induction in CD8+ T cells cocultured with the SCFA-stimulated CRC, we tested whether this cytokine could explain our observations (38, 39). We first added exogenous IFNγ to the CRC cells during their SCFA stimulation and noted that this led to the same increase in surface MHCI on the treated CRC cells as did coculture with the CD8+ T cells (**Figure 2C**). To confirm these observations, we used an IFNGR blocking antibody to inhibit IFNγ signaling in the CRC prior to SCFA stimulation and noted that this almost completely abrogated the upregulation of MHCI on the CRC cell surface (**Figure 2D**). By including the GPR41 inhibitor BHB, we also confirmed that this effect was dependent on the initial stimulation of the CRC cells with butyrate or propionate and could not be achieved by T cells alone (**Figure 1D**). Collectively, this work suggests a two-step activation process where initial stimulation of the CRCs with butyrate or propionate changes their ability to activate CD8+ T cells while also priming the CRC cells to respond to signals emitted by the activated T cells.

### SCFAs upregulate MHCI, antigen processing machinery and chemokines especially in MSI CRC cells

**Figure 3:**
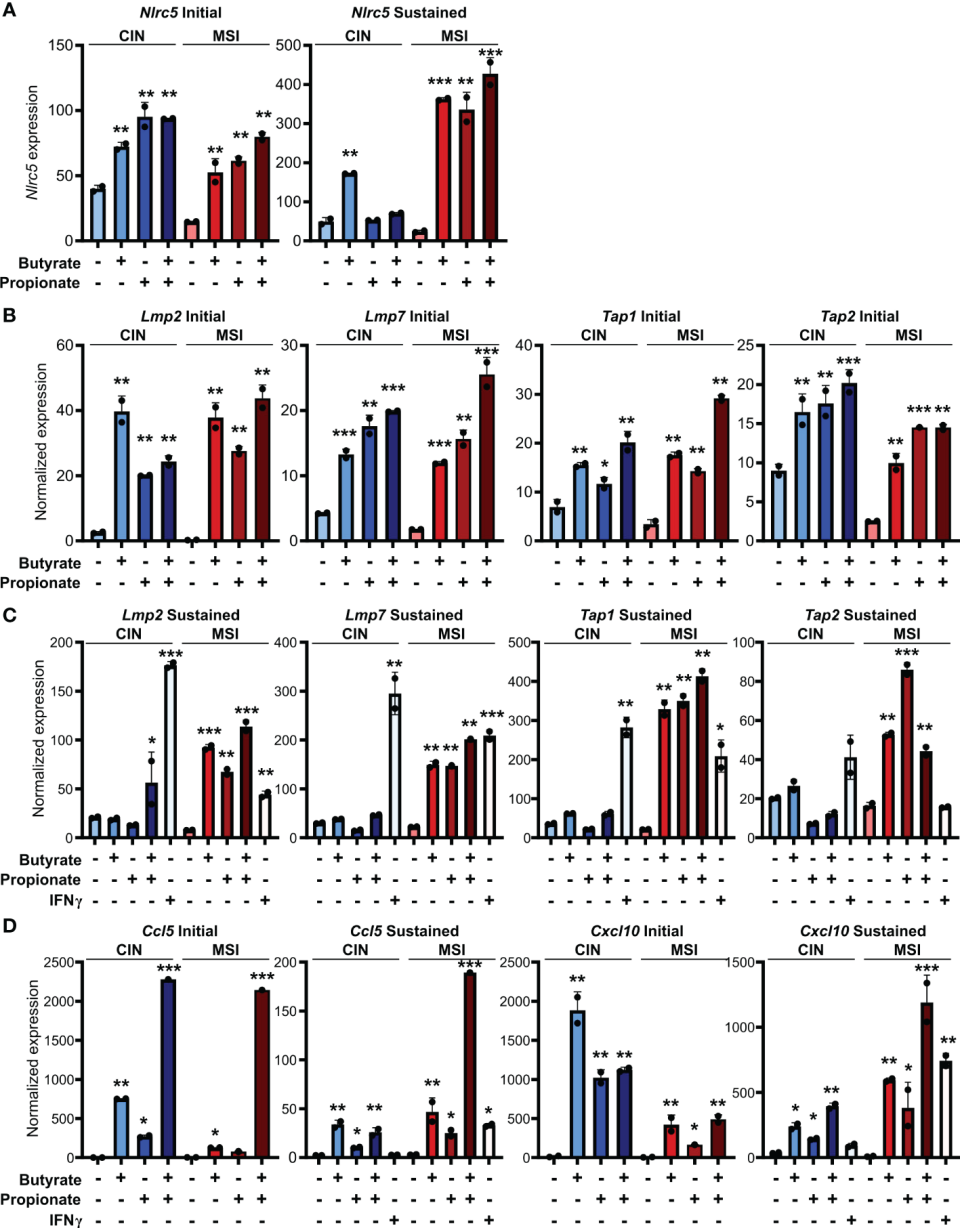
SCFAs increase CRC MHCI antigen processing and presentation machinery most strongly in MSI CRCs. MSI^OVA^ and CIN^OVA^ CRC cells were treated with 50 mM butyrate, 50 mM propionate or a combination of the two for 24 h. CRC cells were then harvested immediately (**A, B, D**: “Initial”) or cultured for a further 24 h in the absence of any treatment (**A, C, D**: “Sustained”). 100 U/ml IFNγ was included with the initial SCFA treatment where indicated. Gene expression was then analyzed by qPCR. For all panels, n = 3 experimental repeats with 2 biological replicates per experiment. Representative graphs from a single experiment are shown. For all panels, relative to the untreated control: *p ≤ 0.05, **p ≤ 0.01, ***p ≤ 0.001.

Our observation of increased MHCI induction on the surface of CRC cells treated with butyrate or propionate could be explained by SCFAs either promoting MHCI biogenesis, MHCI trafficking or overall antigen processing in the CRCs. Since we observed increased total MHCI, including both surface and intracellular protein, we concluded that SCFAs must be upregulating overall MHCI expression rather than simply acting on trafficking. Given that the stability of the MHCI complex depends on antigen loading, butyrate and propionate could either be increasing MHCI synthesis or increasing rates of antigen loading onto MHCI, thereby stabilizing the complex (40, 41). We thus examined whether butyrate and propionate stimulation changed expression of genes involved in the biogenesis and/or loading of MHCI. NLRC5 is a primary transcriptional regulator of MHCI-associated genes and we found its expression to be highly upregulated in CRC cells following treatment with butyrate or propionate (**Figure 3A**) (42–45). Interestingly, although NLRC5 is well known to be regulated by IFNγ, its upregulation in CRC cells was independent of IFNγ exposure and did not require the presence of CD8+ T cells. While it’s possible that *Nlrc5* gene expression could be further enhanced by addition of exogenous IFNγ, our data show this is not necessary and indicate that it is directly induced by the SCFAs. In addition, key genes associated with the trafficking and loading of MHCI such as *Tap1*, *Tap2*, *Lmp2*, *Lmp7* are also highly upregulated by butyrate or propionate treatment independently of IFNγ treatment or CD8+ T cells (**Figures 3B, C**) (46–48). These data suggest that upregulating MHCI antigen presentation in CRCs may be the mechanism underlying initial activation of CD8+ T cells and the onset of the positive feedback loop. Although we initially observed similar levels of induction for these genes in both MSI^OVA^ and CIN^OVA^ CRCs following a 24 h treatment with SCFAs, we discovered that the increased expression was only stable in MSI CRCs where it persisted for 24 h after removal of butyrate or propionate (**Figures 3A–C**).

We had previously shown that MSI CRCs express higher levels of the chemokines CCL5 and CXCL10 and that they are critical to the successful antitumor response in MSI CRCs (16). We thus examined whether SCFAs could influence production of these or other Type I IFN Stimulated Genes (ISGs). Consistent with our previous observations, SCFA treatment led CRC cells to upregulate numerous ISGs, particularly *Ccl5* and *Cxcl10* (**Figure 3D**). As seen with expression of the antigen presentation machinery, this was initially induced to a similar level in all CRCs but was sustained only in MSI CRCs (**Figure 3C**). These finding suggest that the apparent increased sensitivity of MSI CRC cells to SCFAs may contribute to their stronger immunogenicity and overall more favorable prognosis.

### SCFAs induce DNA damage and activate cGAS/STING signaling in CRC cells

**Figure 4:**
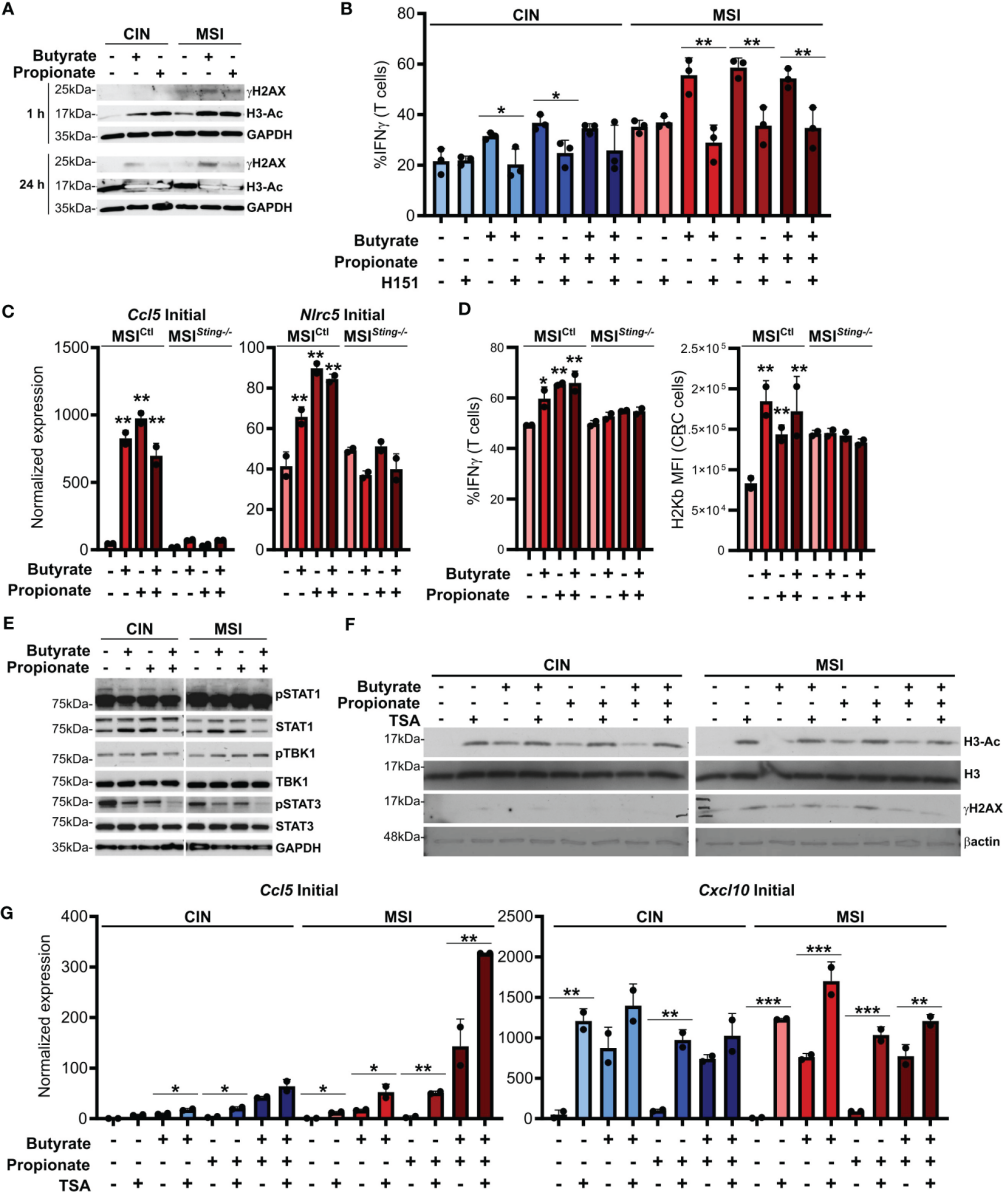
SCFAs induce DNA damage in CRC cells that promotes cGAS/STING signaling and CD8+ T cell activation. **(A)** MSI^OVA^ and CIN^OVA^ CRC cells were treated with 50 mM butyrate, 50 mM propionate or a combination of the two for 1 h or 24 h before harvesting proteins for analysis. **(B)** CRC cells were stimulated as in **(A)** for 24 h in the presence or absence of 10 μM H151. SCFAs were washed off and OVA-specific OTI CD8+ T cells were then added and cocultured with the CRC cells for 24 h before measuring T cell IFNγ production. **(C)** *Sting* was knocked down in MSI CRC cells (MSI*^Sting-\-^*) and compared to a Scramble control (MSI*^Ctl^*). Cells were treated with 50 mM butyrate, 50 mM propionate or a combination of the two for 24 h before harvesting RNA for qPCR analysis. **(D)** MSI*^Sting-/-^* and MSI*^Ctl^* CRC cells were stimulated as in **(C)** for 24 h SCFAs were washed off, cells were pulsed with 1 µg/ml SIINFEKL peptide, and OVA-specific OTI CD8+ T cells were then added and cocultured with the CRC cells for 24 h before measuring T cell IFNγ production or surface CRC H2Kb expression. **(E)** CRC cells were treated as in **(A)** for 24 h before harvesting for protein analysis. **(F)** CRC cells were treated as in **(A)** for 24 h in the presence or absence of 1 μM TSA. **(G)** CRC cells were treated as in **(A)** in the presence or absence of 1 μM TSA. Cells were then harvested immediately (“Initial”) or cultured for an additional 24 h in the absence of additional treatments (“Sustained”). 100 U/ml IFNγ was included with the initial SCFA treatment as indicated. Gene expression was then analyzed by qPCR. For all panels, n = 3 experimental repeats with 2-3 biological replicates per experiment. Representative graphs from a single experiment are shown. For panels **(B, G)**: *p ≤ 0.05, **p ≤ 0.01, ***p ≤ 0.001. For panels **(C, D)** relative to the untreated control: *p ≤ 0.05, **p ≤ 0.01.

ISGs are induced by activation of the cGAS/STING cytosolic DNA sensing pathway. Cancer cells are known to sometimes leak endogenous DNA into the cytosol and this is enhanced by high levels of genetic instability (49, 50). SCFAs have been reported to induce DNA damage and we observed that this occurred to a higher degree in MSI compared to CIN CRCs (**Figure 4A**). This is consistent with the greater underlying genetic instability in these CRCs and we suspected that it might trigger increased activation of cGAS/STING. To test this, we first added the STING inhibitor H151 to the CRC cells in combination with butyrate and propionate for 24 h (51). Following extensive washing to remove both the SCFAs and inhibitor, we added OVA-specific OTI CD8+ T cells and cocultured them with the treated MSI^OVA^ and CIN^OVA^ CRCs for 24 h. STING inhibition significantly decreased both MHCI surface expression on the CRC cells as well as CD8+ T cell activation, strongly indicating that this signaling pathway is one of the mechanisms by which SCFAs promote antitumor immunity in CRCs (**Figure 4B**). In order to confirm this further, we knocked down *Sting* expression in the MSI CRCs (MSI*^Sting-/-^*) and evaluated their response to SCFA stimulation. The *Sting* deficient cells expressed fewer ISGs, activated fewer OVA-specific CD8+ T cells and upregulated less surface MHCI than the *Sting*-expressing scramble MSI*^Ctl^* control cells in response to the SCFAs (**Figures 4C, D**). Given the higher baseline activation we and others have previously reported for cGAS/STING signaling in MSI CRCs, our findings here suggest that this DNA sensing pathway is contributing to the greater sensitivity of MSI CRCs to SCFAs (16, 52).

Somewhat puzzlingly, we did not observe consistent activation of the canonical STING downstream mediators TBK1 and STAT1 in CRC cells treated with SCFAs (**Figure 4E**). Since DNA damage did not become apparent until at least one hour after SCFA treatment of the CRCs and persisted for at least 24 h, especially in the MSI CRC cells, our data suggests that SCFAs do not directly activate the cGAS/STING and do not cross-talk with this pathway directly. Instead, our findings are consistent with a model where SCFAs initially inhibit histone deacetylases, thereby promoting chromosome decondensation (53–55). This in turn increases the susceptibility of the DNA to damage, leading to escape of some endogenous DNA into the cytoplasm where cGAS/STING can become activated. In support of this, DNA acetylation occurs rapidly after SCFA stimulation and precedes the onset of increased DNA damage (**Figure 4A**). Furthermore, stimulation of CRC cells with the HDAC inhibitor trichostatin A (TSA) also induces DNA damage and increases expression of ISGs to a similar extent as do butyrate and propionate (**Figures 4F, G**) (56). Notably, stimulation of CRC cells with both SCFAs and TSA does not lead to further increases, indicating that they both use a common mechanism to upregulate expression of these immunogencity promoting molecules.

### MSI CRC cells strongly express gene signatures associated with butyrate responsiveness and histone acetylation in in vivo models and human CRC patients

**Figure 5:**
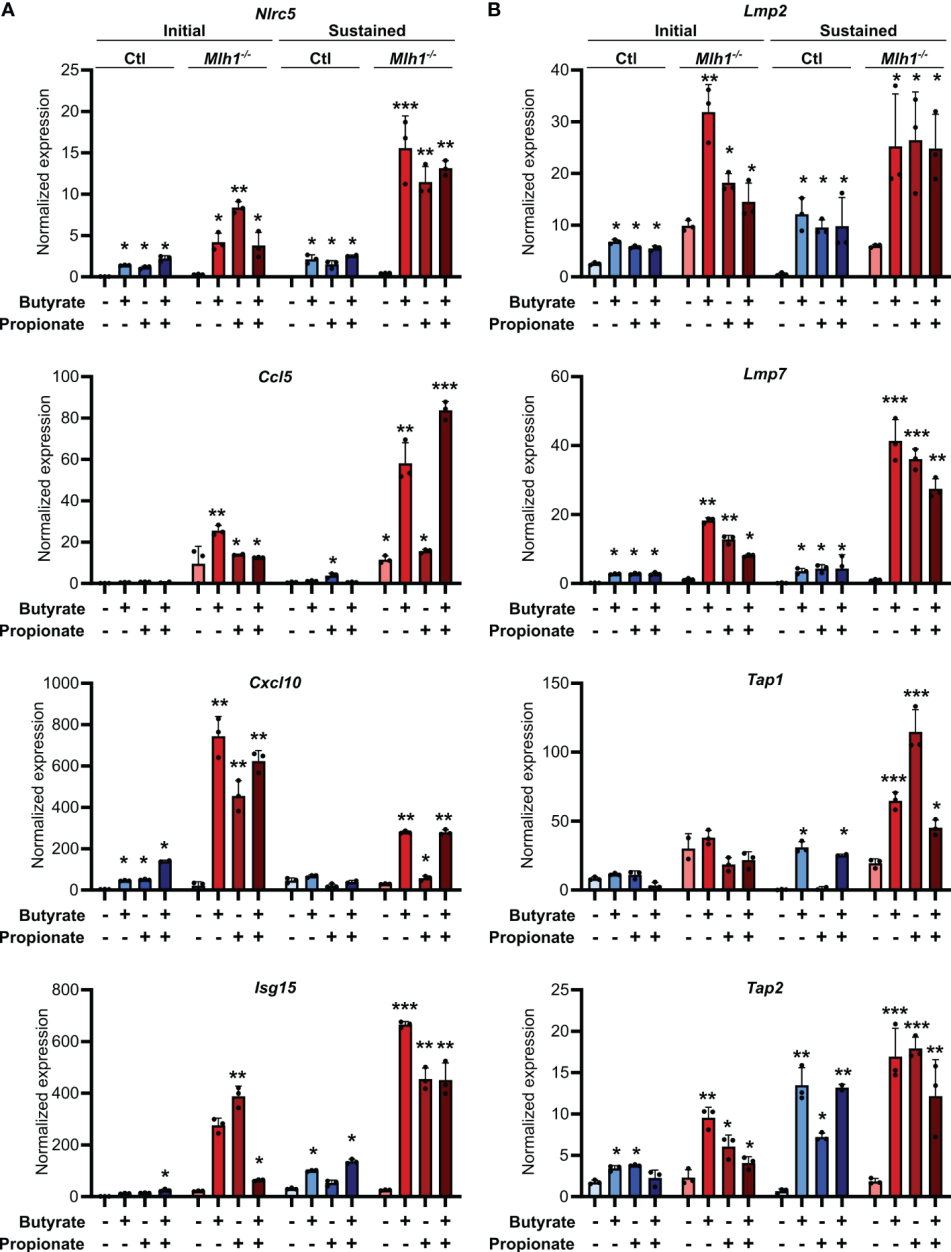
SCFAs upregulate the antigen presenting capacity of primary mouse CRC organoids. Primary CRC organoids were derived from tumors induced by repeated doses of azoxymethane (10 weekly doses of 10 mg/kg azoxymethane) to wild type C57BL/6 mice. *Mlh1* was then knocked down by stably transducing with shRNA (*Mlh1^-/-^*) to create an MSI variant. CIN variants were made using a scrambled sequence (Ctl). Organoids were then treated with 50 mM butyrate, 50 mM propionate or a combination of the two for 24 h. Organoid cells were harvested immediately after the stimulation (“Initial”) or were cultured a further 24 h in the absence of any treatment (“Sustained”). Expression of ISGs **(A)** and antigen processing and presentation machinery **(B)** were the analyzed by qPCR. For all panels, n = 3 experimental repeats with 3 biological replicates per experiment. Representative graphs from a single experiment are shown. For all panels, relative to the untreated control: *p ≤ 0.05, **p ≤ 0.01, ***p ≤ 0.001.

Our experiments consistently showed that MSI CRCs have an increased sensitivity to the immune stimulatory properties of butyrate and propionate. However, these experiments were based in a single cell system, and we thus sought to validate our findings in a more physiologically relevant one. We first used primary CRC organoids derived from an *Apc^Min/+^* mouse and in which we had stably knocked down *Mlh1* using shRNA to generate an MSI variant (*Mlh1^-/-^*) (16, 57). Stimulation of these organoids with butyrate and propionate upregulated both CCL5 and CXCL10 as well as many molecular mediators of antigen presentation (**Figure 5**). Consistent with our previous findings, the effect was sustained for a prolonged period in the *Mlh1^-/-^* MSI variant, thereby confirming their greater susceptibility to SCFA-induced antitumor immunity.

**Figure 6:**
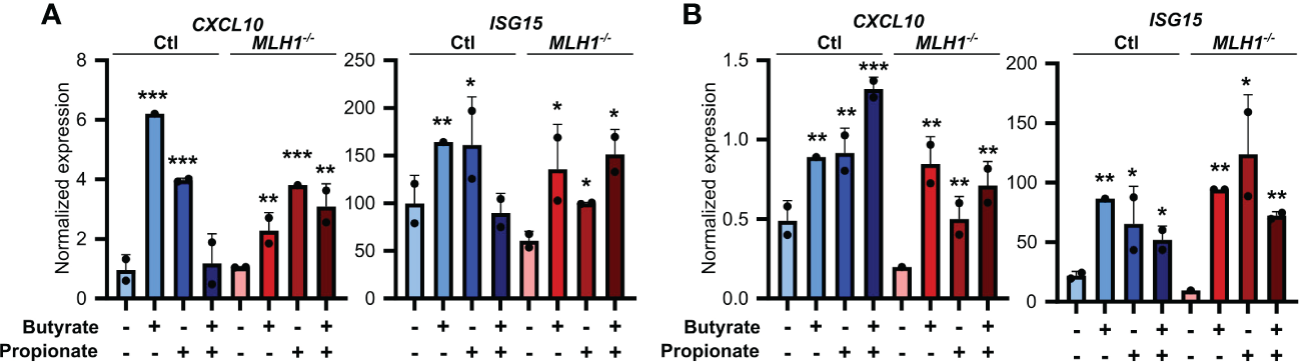
SCFAs upregulate the antigen presenting capacity of primary CRC patient organoids. Primary CRC patient organoids were derived from two separate patients, CRC-A **(A)** and CRC-B **(B)**. For organoids from each patient, *MLH1* was then knocked down by stably transducing with shRNA (*MLH1^-/-^*) to create an MSI variant. CIN variants were made using a scrambled sequence (Ctl). Organoids were then treated with 25 mM butyrate, 25 mM propionate or a combination of the two for 24 h. Organoid cells were harvested immediately after the stimulation and gene expression was analyzed by qPCR. For all panels, n = 3 experimental repeats with 2 biological replicates per experiment. Representative graphs from a single experiment are shown. For all panels, relative to the untreated control: *p ≤ 0.05, **p ≤ 0.01, ***p ≤ 0.001.

**Figure 7:**
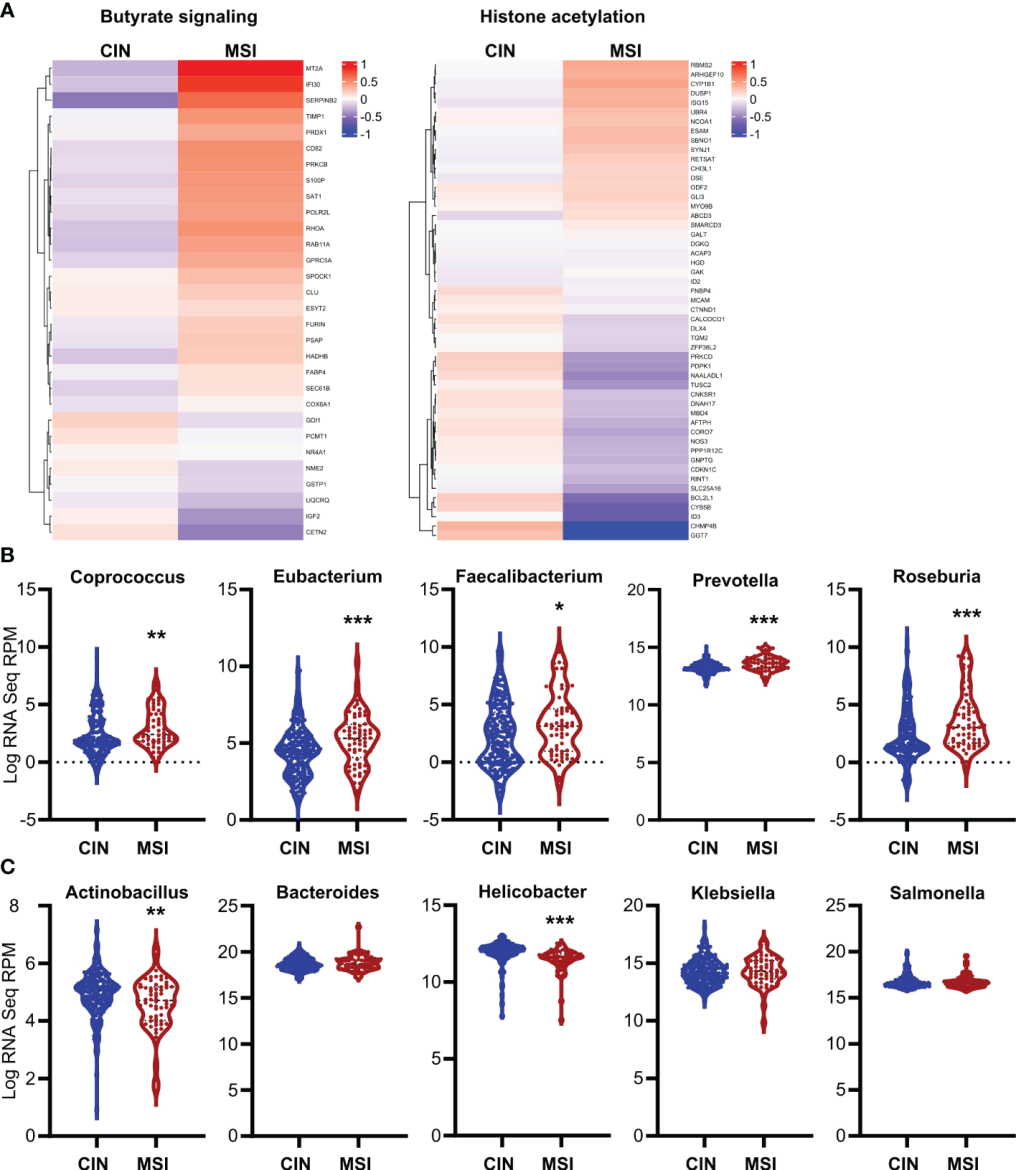
Human MSI CRCs more strongly express gene signatures associated with butyrate signaling and are enriched in butyrate producing microbial taxa. **(A)** CRC data from the PanCancer dataset in The Cancer Genome Atlas (TCGA) were analyzed for expression of genes associated with GSEA signatures for butyrate signaling and histone acetylation. **(B, C)** Microbial signatures from the TCGA CRC PanCancer dataset were analyzed for the most common butyrate producing taxa **(B)** or for other non-butyrate producing taxa commonly associated with CRC **(C)**. *p ≤ 0.05, **p ≤ 0.01, ***p ≤ 0.001.

To ensure the relevance of our findings to human CRC patients, we first made organoids from two CRC patients and generated an MSI variant of each by knocking down *MLH1* (*MLH1^-/-^*). Stimulation of these organoids with SCFAs upregulated several ISGs and did so more strongly in the *MLH1^-/-^* MSI variant of each patient’s organoids compared to the control CIN variant (Ctl) (**Figure 6**). We next used a broader approach by examining data from the CRC tumors in The Cancer Genome Atlas (TCGA) PanCancer dataset (29, 31, 58, 59). We identified genesets associated with increased butyrate signaling and histone acetylation using the MSigDB Gene Ontology (GO) resource and analyzed their expression levels in MSI and CIN CRCs (26). As shown in **Figure 7A**, MSI CRC tumors express higher levels of the genes associated with butyrate signaling and histone acetylation, suggesting that human MSI CRCs do possess a greater sensitivity to SCFAs. This finding could, however, also result from a higher level of butyrate in the intestine of MSI CRC patients if they have an enrichment of SCFA-producing bacteria. We thus examined the microbial signatures associated with the CRC tumors in the PanCancer dataset and noted MSI CRCs had greater amounts of all of the predominant butyrate producing taxa (**Figure 7B**) but not of non-butyrate producing taxa that are frequently associated with CRC (**Figure 7C**).

**Figure 8:**
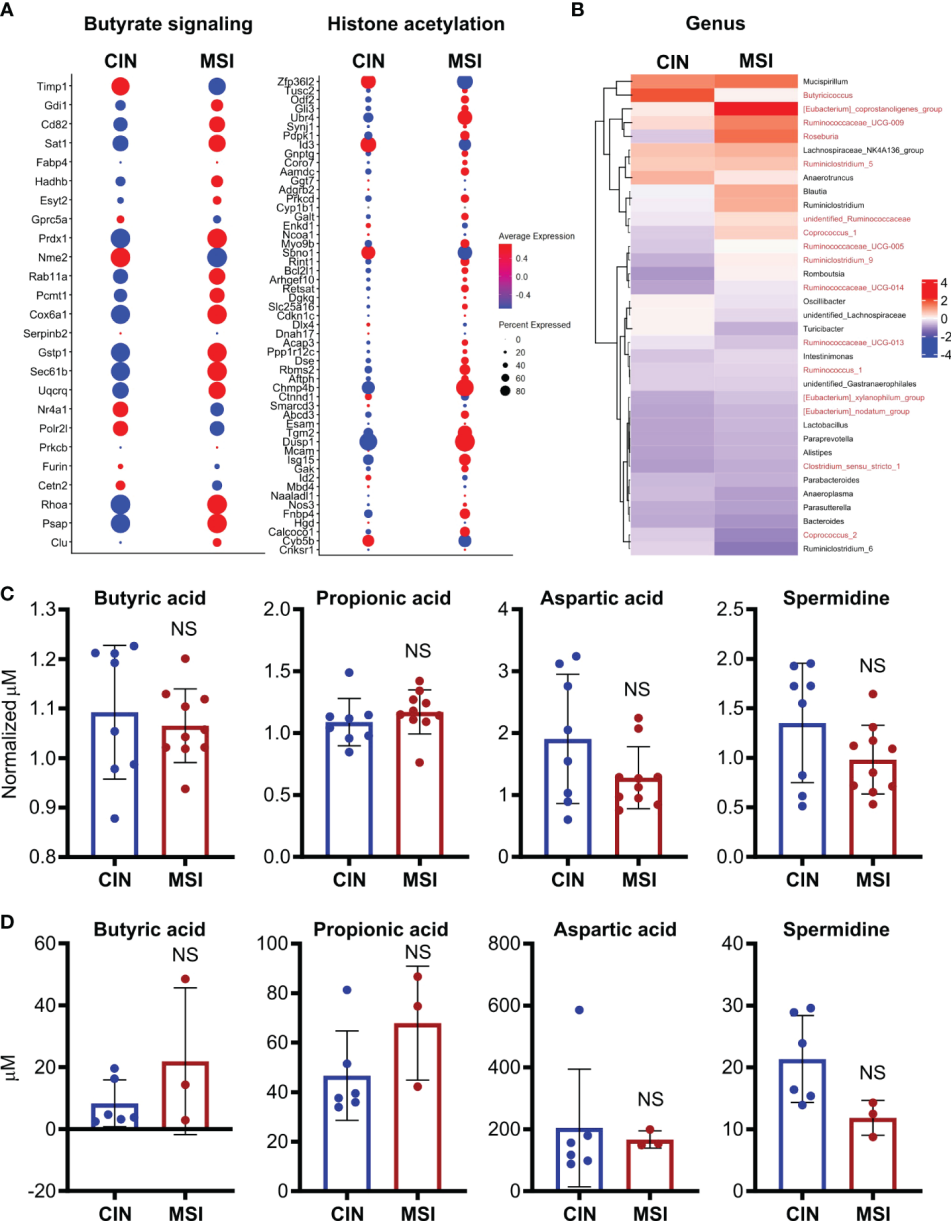
Orthotopic MSI CRCs are more sensitive to the immune-promoting effects of SCFAs independently of the amount of SCFA production by their microbiota. **(A)** scRNAseq was performed on MSI and CIN CRCs grown orthotopically in the colon following endoscopic implantation. 5 mice were pooled from each CRC type. Expression of genes associated with GSEA signatures of butyrate signaling and histone acetylation were analyzed. **(B)** 16S rRNA sequencing was performed on the feces of mice implanted with orthotopic MSI and CIN CRCs. Relative abundance of the indicated microbial genera are presented. The names of butyrate producing taxa are colored in red. n = 2 repeats, 6 mice total per group. **(C, D)** Metabolite profiling was performed on the feces **(C)** and tumor tissue **(D)** from mice implanted with orthotopic MSI and CIN CRCs. The abundance of SCFAs is presented as concentrations and, for the feces, the values are normalized to the abundance of SCFAs in fecal samples from the mice taken before tumor implantation. No significant differences were found in SCFA expression between MSI and CIN samples. n = 2 repeats. scRNAseq data in panel **(A)** were from the dataset GSE178706 at the NCBI Gene Expression Omnibus which we previously published (16). 16S rRNA sequencing and metabolomics data in panels **(B–D)** were newly generated.

To better elucidate the relationship between MSI CRCs, SCFAs and antitumor immunity, we used an orthotopic in vivo system where our MSI and CIN MC38 CRC cells were injected endoscopically in the colons of immunocompetent wild type mice (16). Using a previously published scRNAseq dataset generated from such orthotopically grown tumors, we examined expression of genesets for butyrate signaling and histone acetylation specifically in the CRC cells. This revealed that MSI CRCs also expressed more of the genes associated with these two pathways (**Figure 8A**). We next performed 16S rRNA sequencing on the feces of mice bearing orthotopic MSI and CIN CRCs to determine if differences in the microbial environment could account for our observations. We observed an enrichment of the predominant butyrate producing taxa in mice with MSI CRCs, indicating that they might have higher amounts of SCFAs in the intestine that could account for their greater expression of butyrate signaling genes (**Figure 8B**) (60, 61). We thus performed a metabolomics screen of the feces and tumor tissue from orthotopic MSI and CIN CRC-bearing mice. Surprisingly, we did not identify any differences in the amount of SCFA metabolites in either the feces (**Figure 8C**) or tumor tissue (**Figure 8D**) of MSI and CIN CRC bearing mice. This finding supports our hypothesis that MSI CRCs have a higher baseline sensitivity to SCFAs and that this is one mechanism by which they induce successful antitumor immune responses.

## Discussion

CRC is one of the top three causes of cancer-related death worldwide and the contribution of microbiota to its pathogenesis is increasingly being recognized (62). Clear evidence has identified mechanisms by which microbial products, such as the SCFAs butyrate and propionate, can directly contribute to the transformation of intestinal epithelial cells, mutagenesis of the CRC genome and alterations in CRC cell proliferation and metabolism (1, 3). A further large body of evidence has documented the potent immune regulating potential of these metabolites, including strong immunosuppressive effects of SCFAs directly on many immune cells (6). However, little is known about how SCFAs change the ability of CRC cells to regulate antitumor immunity. This represents a critical knowledge gap given that intestinal epithelial cells are exposed to much higher concentrations of SCFAs than the underlying intestinal immune cells and are thus positioned as front-line mediators of SCFA-mediated immune regulation. We demonstrate here that SCFAs also directly and potently regulate the immunogenicity of CRC cells and that the ultimate outcome of this depends on the underlying genotype of the CRC cells. Specifically, direct stimulation of CRC cells with SCFAs upregulates their ability to activate cytotoxic CD8+ T cells but the magnitude of this effect differs according to the CRC subtype and is strongest in those with deficient DNA repair.

Underlying this three-way relationship between SCFAs, CRC cells, and CD8+ T cells is a two-step feedback mechanism where butyrate and propionate directly upregulate genes involved in cytokine production, antigen processing and MHCI generation in CRC cells, all of which contribute to CD8+ T cell activation. The activated CD8+ T cells secrete high amounts of IFNγ which then feeds back on the cancer cells to further upregulate CRC cell MHCI, further increasing their capacity to activate CD8+ T cells. This feedback loop is stronger and more sustained in cancers with defective DNA mismatch repair and higher genetic instability. This is supported by our observation that butyrate and propionate induced greater DNA damage in the DNA repair deficient MSI CRC cells, which are typically quite immunogenic. Although such increased DNA damage could be expected to promote tumorigenesis, as has been speculated by previous studies documenting SCFA-induced changes to CRC DNA repair, our findings indicate that, in some CRCs, this may be counterbalanced by an increased antitumor immune response (7, 9–12). Indeed, numerous reports indicate that SCFAs can sometimes decrease DNA damage in CRCs, making it clear that further study is needed to understand how both the underlying CRC genome and surrounding tumor microenvironment alter the ultimate outcome of SCFA stimulation of CRC cells (63–65). Included in the latter is likely the composition and functional output of a CRC patient’s intestinal microbiome since cancer is often associated with an increase of the major butyrate producing taxa, *Firmicutes*, coupled with decreased levels of *Proteobacteria* (60, 61, 66, 67). Indeed, both our mouse and human data indicated higher amounts of the main butyrate-producing taxa in MSI compared to CIN CRCs. However, we did not find higher amounts of the SCFAs themselves in either the feces or tumors of the MSI CRC-bearing mice. Collectively, our data is consistent with the fact that SCFAs promote anti-tumor immunity in both MSI and CIN CRC cells but that the MSI CRC cells are more sensitive to this effect. The stronger anti-tumor immune response associated with MSI CRCs may thus be promoted both by their greater sensitivity to SCFAs as well as by an enrichment of butyrate-producing taxa within their microbiota. Further research will be needed to understand the relative contribution of each of these factors.

Central to resolving this complex picture is undoubtedly to better understand the mechanisms by which SCFAs such as butyrate and propionate mediate their immune-regulating effects directly on the CRCs. While we have used BHB to show that the main butyrate and propionate receptors contribute to the process, BHB exerts many other effects on cells besides GPR41 inhibition (35). In the absence of more specific inhibitors for the highly homologous SCFA receptors, it is difficult to show conclusively that signaling via these receptors directly induces DNA damage and drives the first step in the feedback loop (34, 68). Instead, we speculate that the main mechanism by which SCFAs initiate improved antitumor immunity in CRC cells is via their function as HDAC inhibitors. Decondensing chromosomes is known to change susceptibility of DNA to potentially damaging agents and to alter the efficacy of DNA repair at the newly exposed sites. We believe this occurs in response to SCFAs that block deacetylation of histones, leading to greater DNA damage and genetic instability. Our data show that this in turn activates cGAS/STING in the CRC cells which we have already shown to be essential for induction of antitumor immunity in MSI CRCs (16). This is supported by our demonstration that the HDAC inhibitor TSA phenocopies the immune effects of butyrate and propionate stimulation on CRCs (69, 70). This suggests that part of the efficacy of HDAC inhibitors in clinical trials results from regulation of the tumor cells’ own ability to promote an antitumor immune response.

Further study is needed to investigate this possibility and more clearly delineate the underlying mechanisms. Understanding the role of the SCFA receptors will require development of more specific inhibitors for each of the highly homologous SCFA receptors (34). In addition, truly understanding the role of HDAC inhibition in SCFA-regulated antitumor immunity in CRC cells necessitates better delineation of which HDACs are regulated by specific SCFAs or the identification of inhibitors for specific histone acetyltransferases that can counteract the actions of SCFAs. Our finding that both butyrate and propionate induce similar immune effects on CRC cells indicates that this may be a general mechanism shared by many SCFAs and that a better understanding of the differences between them could identify ways of boosting CRC-mediated antitumor immunity without the important risk of simultaneously inhibiting activation of infiltrating immune cells. A more in depth study that uses matched samples of tumor cells, immune cells and feces from the same CRC patients with different dysregulated DNA repair pathways would also be highly valuable in better understanding which of these is the dominant driver of the relationship between SCFAs and antitumor immunity.

It is now widely accepted that stimulating antitumor immunity is one of the most promising strategies for treating cancer patients and finding additional strategies to do so can improve the performance of existing therapies in addition to helping develop new ones. Our findings demonstrate that SCFAs, or compounds that mimic their effects, are a promising therapeutic avenue to augment antitumor immunity in CRC patients, especially those with MSI CRCs. Although further pre-clinical work is needed to validate and extend our findings, SCFAs could be applied to cancer therapy in several ways. In CIN CRC patients, administration of SCFAs in conjunction with DNA damaging agents could improve patient outcome while minimizing toxicity. The presence or absence of SCFA-producing taxa in a patient’s intestinal flora could also help predict the likelihood of a CRC patient responding to immune-based therapies such as anti-PD1/PDL1. Deepening our understanding of how SCFAs regulate the immunogenicity of CRCs thus offers multiple opportunities to improve current and future patient care.

## Data Availability

The datasets presented in this study can be found in online repositories. The names of the repository/repositories and accession number(s) can be found below: SAMN34441860-SAMN34441871 (NCBI-GenBank).
