# Correction: Short chain fatty acids prime colorectal cancer cells to activate antitumor immunity

**DOI:** 10.3389/fimmu.2025.1700962

**Published:** 2025-09-17

**Authors:** Courtney Mowat, Jasmine Dhatt, Ilsa Bhatti, Angela Hamie, Kristi Baker

**Affiliations:** ^1^ Department of Oncology, University of Alberta, Edmonton, AB, Canada; ^2^ Department of Medical Microbiology and Immunology, University of Alberta, Edmonton, AB, Canada

**Keywords:** colorectal cancer, antitumor immunity, microbiota, short chain fatty acid (SCFA), microsatellite instability, HDAC

It has come to our attention that one of the papers cited in our publication has been retracted. We would thus like to remove the following citation from our manuscript.

“Poore GD, Kopylova E, Zhu Q, Carpenter C, Fraraccio S, Wandro S, et al. Microbiome analyses of blood and tissues suggest cancer diagnostic approach. *Nature* (2020) 579(7800):567–74. doi: 10.1038/s41586-020-2095-1”

The original version of this article has been updated.

