# Supplementary material for: Short chain fatty acids prime colorectal cancer cells to activate antitumor immunity
Source: Front Immunol. 2023 May 25;14:1190810. doi: 10.3389/fimmu.2023.1190810 (PMC10248408; doi:10.3389/fimmu.2023.1190810)
Supplement: Supplementary file 1 [file Table1.docx]

**Supplementary Files**

**Table S1. Primers**

| Primer Name | Primer Sequence (5’🡪3’) | Purpose |
| --- | --- | --- |
| *Mlh1* A gRNA Forward | caccgGACGGTAGTGAACCGCATAGCGG | CRISPR |
| *Mlh1* A gRNA Reverse | aaacCCGCTATGCGGTTCACTACCGTCc | CRISPR |
| *Mlh1* B gRNA Forward | caccgGGTAGTGAACCGCATAGCGGCGG | CRISPR |
| *Mlh1* B gRNA Reverse | aaacCCGCCGCTATGCGGTTCACTACCc | CRISPR |
| *Kras* A gRNA Forward | caccgGCCGCCTGCCGAATCGAGCCCGG | CRISPR |
| *Kras* A gRNA Reverse | aaacCCGGGCTCGATTCGGCAGGCGGCc | CRISPR |
| *mMlh1*_078_shRNA Forward | AATTGCAGGTATTCAATACACAATTCTCGAGAATTGTGTATTGAATACCTGCTTTTTTTAT | shRNA |
| *mMlh1*_078_shRNA Reverse | AAAAAAAGCAGGTATTCAATACACAATTCTCGAGAATTGTGTATTGAATACCTGC | shRNA |
| *hMLH1*_053_shRNA Forward | AATTGTGTTCTTCTTTCTCTGTATTCTCGAGAATACAGAGAAAGAAGAACACTTTTTTTAT | shRNA |
| *hMLH1*_053_shRNA Reverse | AAAAAAAGTGTTCTTCTTTCTCTGTATTCTCGAGAATACAGAGAAAGAAGAACAC | shRNA |
| *Sting*_shRNA_Forward | GCATCAAGAATCGGGTTTATT | shRNA |
| *Sting*_shRNA Reverse | CAACATTCGATTCCGAGATAT | shRNA |
| M13/pUC Forward | CCCAGTCACGACGTTGTAAAACG | Sequencing |
| M13/pUC Reverse | AGCGGATAACAATTTCACACAGG | Sequencing |
| *Mlh1* Seq Forward | GCGCGCGAATTCCCAAATCAAATGTCCGAGGGC | Sequencing |
| *Mlh1* Seq Reverse | GCGCGCGGATCCGTAGCAGGAGTTATTCGGCGT | Sequencing |
| *Ccl5* Forward | GCTGCTTTGCCTACCTCTCC | qPCR |
| *Ccl5* Reverse | TCGAGTGACAAACACGACTGC | qPCR |
| *Cxcl10* Forward | CCAAGTGCTGCCGTCATTTTC | qPCR |
| *Cxcl10* Reverse | GGCTCGCAGGGATGATTTCAA | qPCR |
| *Isg15* Forward | GGTGTCCGTGACTAACTCCAT | qPCR |
| *Isg15* Reverse | TGGAAAGGGTAAGACCGTCCT | qPCR |
| *Lmp2* Forward | AAGTCCACACCGGGACAAC | qPCR |
| *Lmp2* Revers | TTCTTCACCACGTTTGCAGC | qPCR |
| *Lmp7* Forward | GCCAAGGAGTGCAGGTTGTAT | qPCR |
| *Lmp7* Reverse | GCCGAGTCCCATTGTCATCT | qPCR |
| *Tap1* Forward | TGGGAAAAGTGCATCAGTCAC | qPCR |
| *Tap1* Reverse | AATGAGACAAGGTTGCCGCTG | qPCR |
| *Tap2* Forward | TGTGAGGACGCTCAAGTGAT | qPCR |
| *Tap2* Reverse | CTCCAGTTCTGTAGGGCCTGT | qPCR |
| *Nlrc5* Forward | TTGCCCAGAAACTGGACCTC | qPCR |
| *Nlrc5* Reverse | CACGGTGTTGTTTAGCAGGC | qPCR |
| *Gapdh* Forward | CATGTTCCAGTATGACTCCA | qPCR |
| *Gapdh* Reverse | TGAAGACACCAGTAGACTCC | qPCR |

**Table S2. Antibodies**

| Target | Fluorophore or Secondary | Purpose | Source |
| --- | --- | --- | --- |
| b-Actin | Anti-rabbit IgG HRP | Western Blot | Cell Signaling (8457S) |
| GAPDH | Anti-mouse IgG HRP | Western Blot | ThermoFisher (PIMA515738) |
| Phospho-TBK1 (Ser172) | Anti-rabbit IgG HRP or Alexa 488 | Western Blot, Flow Cytometry | Cell Signaling (5483S) |
| TBK1 | Anti-rabbit IgG HRP | Western Blot | Cell Signaling (3504S) |
| Phospho-STAT1 (Tyr701) | Anti-rabbit IgG HRP | Western Blot | Cell Signaling (7649S) |
| STAT1 | Anti-rabbit IgG HRP | Western Blot | Santa Cruz (sc-464) |
| Phospho-STAT3 (Tyr705) | Anti-rabbit IgG HRP | Western Blot | Cell Signaling (9131S) |
| STAT3 | Anti-rabbit IgG HRP | Western Blot | Cell Signaling (12640S) |
| γH2AX | Anti-rabbit IgG HRP | Western Blot | Cell Signaling (9718S) |
| H3-Ac | Anti-rabbit IgG HRP | Western Blot | EMD Millipore (06-599) |
| H3 | Anti-rabbit IgG HRP | Western Blot | AbCam (ab1791) |
| CD3 | APCCY7 | Flow Cytometry | Biolegend (100222) |
| CD8A | ALEXA-700 | Flow Cytometry | ThermoFisher (56-0081-82) |
| CD45 | ALEXA-700 | Flow Cytometry | ThermoFisher (56-0451-82) |
| H-2Kb | PE | Flow Cytometry | Biolegend (116507) |
| H-2Kb-SIINFEKL | PE | Flow Cytometry | ThermoFisher (12-5743-82) |
| IFNγ | PE | Flow Cytometry | ThermoFisher (12-7311-82) |
| IFNγ | APC | Flow Cytometry | ThermoFisher (17-7311-82) |
| IFNGR |  | Blocking | BioXcell (BE0029) |
| Mouse IgG | HRP | Western Blot | Cell Signaling 7076S) |
| Rabbit IgG | HRP | Western Blot | Cell Signaling (7074S) |
